# Genetic barrier to resistance: a critical parameter for efficacy of neutralizing monoclonal antibodies against SARS-CoV-2 in a nonhuman primate model

**DOI:** 10.1128/jvi.00628-24

**Published:** 2024-06-20

**Authors:** Christiane Stahl-Hennig, Antonia Sophia Peter, Arne Cordsmeier, Nicole Stolte-Leeb, Ramona Vestweber, Eileen Socher, Samuel Alberto Merida, Ulrike Sauermann, Martina Bleyer, Kirsten Fraedrich, Thomas Grunwald, Thomas H. Winkler, Armin Ensser, Hans-Martin Jäck, Klaus Überla

**Affiliations:** 1 Unit of Infection Models, German Primate Center, Göttingen, Germany; 2 Institute of Clinical and Molecular Virology, Universitätsklinikum Erlangen, Friedrich-Alexander-Universität Erlangen-Nürnberg, Erlangen, Germany; 3 Institute of Anatomy, Functional and Clinical Anatomy, Friedrich-Alexander-Universität Erlangen-Nürnberg, Erlangen, Germany; 4 Department of Vaccines and Infection Models, Fraunhofer Institute for Cell Therapy and Immunology IZI, Leipzig, Germany; 5 Division of Genetics, Department Biology, Nikolaus-Fiebiger-Center of Molecular Medicine, Friedrich-Alexander-Universität Erlangen-Nürnberg, Erlangen, Germany; 6 Division of Molecular Immunology, Internal Medicine III, Nikolaus-Fiebiger-Center of Molecular Medicine, Friedrich-Alexander-Universität Erlangen-Nürnberg, Erlangen, Germany; University of North Carolina at Chapel Hill, Chapel Hill, North Carolina, USA

**Keywords:** SARS-CoV-2, nonhuman primate model, neutralizing monoclonal antibodies, barrier to resistance, antibody therapy

## Abstract

The potency of antibody neutralization in cell culture has been used as the key criterion for selection of antibodies against severe acute respiratory syndrome coronavirus 2 (SARS-CoV-2) for clinical development. As other aspects may also influence the degree of protection *in vivo*, we compared the efficacy of two neutralizing monoclonal antibodies (TRES6 and 4C12) targeting different epitopes of the receptor binding domain (RBD) of SARS-CoV-2 in a prophylactic setting in rhesus monkeys. All four animals treated with TRES6 had reduced viral loads in the upper respiratory tract 2 days after naso-oropharyngeal challenge with the Alpha SARS-CoV-2 variant. Starting 2 days after challenge, mutations conferring resistance to TRES6 were dominant in two of the rhesus monkeys, with both animals failing to maintain reduced viral loads. Consistent with its lower serum neutralization titer at the day of challenge, prophylaxis with 4C12 tended to suppress viral load at day 2 less efficiently than TRES6. However, a week after challenge, mean viral loads in the lower respiratory tract in 4C12-treated animals were lower than in the TRES6 group and no mutations conferring resistance to 4C12 could be detected in viral isolates from nasal or throat swabs. Thus, genetic barrier to resistance seems to be a critical parameter for the efficacy of prophylaxis with monoclonal antibodies against SARS-CoV-2. Furthermore, comparison of antibody concentrations in respiratory secretions to those in serum shows reduced distribution of the 4C12 antibody into respiratory secretions and a delay in the appearance of antibodies in bronchoalveolar lavage fluid compared to their appearance in secretions of the upper respiratory tract.

**IMPORTANCE:**

Monoclonal antibodies are a powerful tool for the prophylaxis and treatment of acute viral infections. Hence, they were one of the first therapeutic agents licensed for the treatment of severe acute respiratory syndrome coronavirus 2 (SARS-CoV-2). Oftentimes, the main criterion for the selection of antibodies for clinical development is their potency of neutralization in cell culture. By comparing two antibodies targeting the Spike protein of SARS-CoV-2, we now observed that the antibody that neutralized SARS-CoV-2 more efficiently in cell culture suppressed viral load in challenged rhesus monkeys to a lesser extent. Extraordinary rapid emergence of mutants of the challenge virus, which had lost their sensitivity to the antibody, was identified as the major reason for the reduced efficacy of the antibody in rhesus monkeys. Therefore, the viral genetic barrier to resistance to antibodies also affects their efficacy.

## INTRODUCTION

Monoclonal antibodies (mAbs) neutralizing severe acute respiratory syndrome coronavirus 2 (SARS-CoV-2) were the first antiviral drugs specifically developed for the treatment and prophylaxis of coronavirus disease 2019 (COVID-19) (1). Emergence of variants of concern (VOC) resistant to such antibodies on population level, however, limits their broad applicability. The Omicron variants, for example, are only neutralized by a small subset of monoclonal antibodies developed for the treatment of SARS-CoV-2 infections (2, 3). As more variants emerge, having a repertoire of monoclonal antibodies targeting different sites of the Spike protein may become useful. Therefore, we explored the prophylactic efficacy of two monoclonal antibodies targeting the receptor binding domain (RBD) of SARS-CoV-2 in a nonhuman primate model of SARS-CoV-2 infection. We additionally investigated their genetic barrier to resistance *in vivo* and *in vitro*.

The monoclonal TRES6 antibody has been obtained by immunization of TRIANNI mice harboring the human variable heavy and light chain repertoire with SARS-CoV-2 Spike (4). The fully humanized antibody neutralizes the parental SARS-CoV-2 strain with an 50% inhibitory concentration (IC50) of 33 ng/mL and the Alpha and Beta variant with similar efficiency. Through the generation and analysis of escape mutations to TRES6, it was determined that the Spike amino acids S477 and T478 are part of its target site. The antibody is able to compete for angiotensin converting enzyme 2 (ACE-2) binding with the virus and most likely diminishes infection by blockage of the ACE-2 virus interaction (5, 6).

The 4C12 monoclonal antibody [4C12-B12 in patent (7)] was selected from a human synthetic phage library (8) and targets an epitope centered on the Spike amino acid I468 (7), distant from TRES6 and adjacent to the ACE-2 binding site (6). The M252Y/S254T/T256E mutations were additionally introduced into the Fc fragment of 4C12 (YTE mutation). This modification has previously been shown to enhance antibody half-life *in vivo* (9).

Both antibodies, TRES6 and 4C12, showed efficacy in a prophylactic efficacy study in K18-hACE-2 mice transgenic for human ACE-2. Here, the mice were protected from SARS-CoV-2-associated weight loss and displayed a reduced viral load in the lung and other organs in comparison to isotype control treated animals (data not shown).

To investigate the *in vivo* prophylactic efficacy for these two monoclonal antibodies against the SARS-CoV-2 Alpha VOC in a large animal model, a nonhuman primate study was performed.

## RESULTS

In preparation of the nonhuman primate study, the IC50s of TRES6 and 4C12 were determined in a side-by-side approach using a lentiviral pseudotype assay with the Spike protein of the Alpha variant. This revealed IC50s of 7.8 ng/mL and 41 ng/mL for TRES6 and 4C12, respectively. In addition, the IC50 was determined in a live virus neutralization assay with the isolate of the Alpha variant used for the challenge in the nonhuman primate (NHP) study (TRES6 29.4 ng/mL, 4C12 286.6 ng/mL).

In order to determine the challenge dose of the SARS-CoV-2 Alpha variant, a pilot experiment was conducted in which one rhesus monkey was exposed to 10^5^ 50% tissue culture infectious dose (TCID50) and one to 10^6^ TCID50 of the SARS-CoV-2 Alpha variant. The virus was applied by an intranasal and oropharyngeal spray delivery with CE-marked, small, disposable, mechanic aerosolization devices attached to a conventional syringe. To be able to study spread of the challenge virus from the upper to the lower respiratory tract, the trachea of the anaesthetized animals was blocked by inflation of the cuff of an endotracheal tube during the spray delivery and for 20 min thereafter. Viral loads were determined in nasal and oropharyngeal swabs as well as in bronchoalveolar lavage fluids (BALF), and monkeys were euthanized on day 7 or 8. Infection could readily be detected on day 2 by the presence of viral RNA in the respiratory samples. Viral loads did not differ substantially between the two animals (Fig. S1). Therefore, the lower dose was used for the subsequent passive immunization and challenge study.

**Fig 1:**
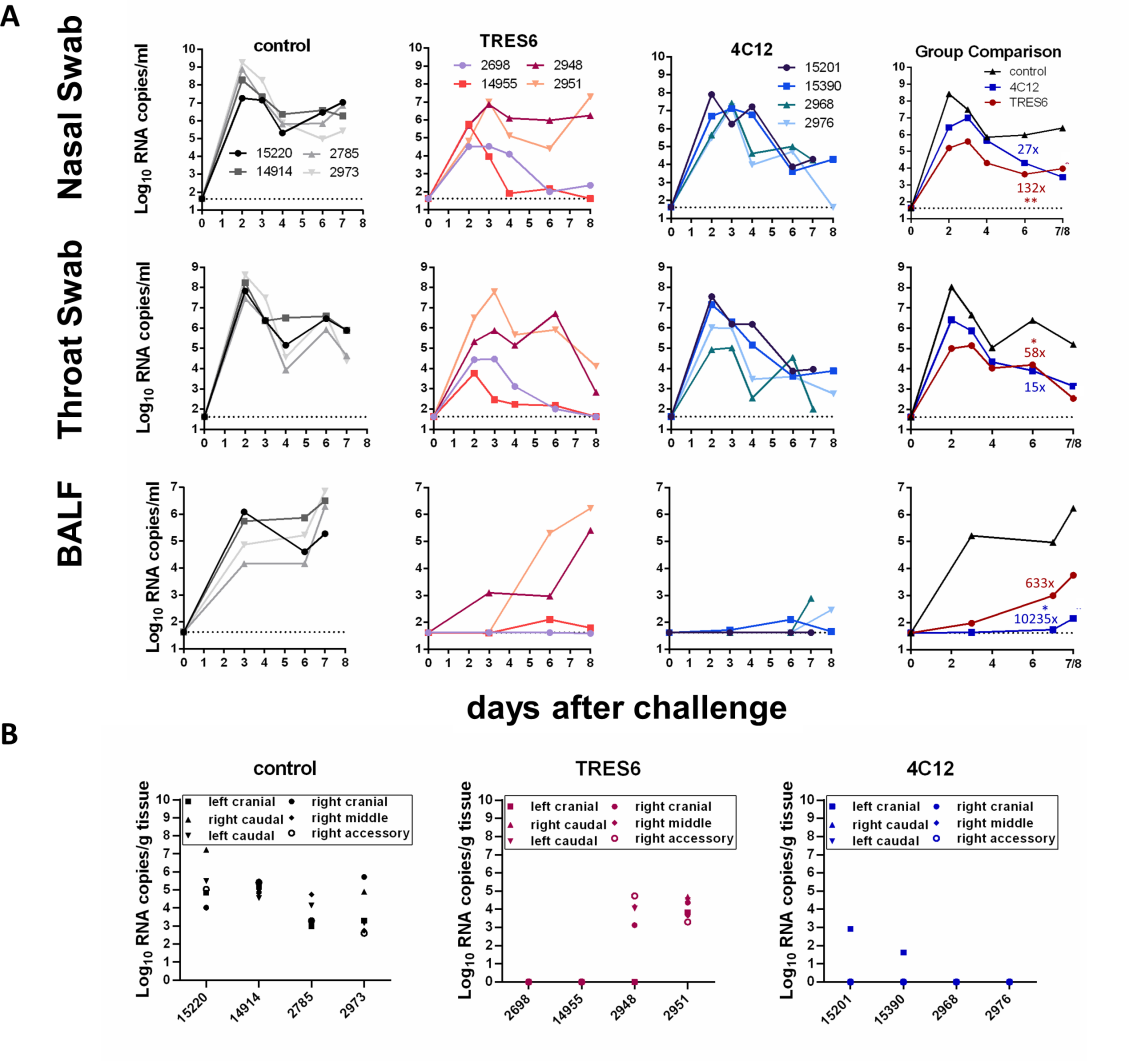
Viral RNA load in respiratory secretions and lung lobes of TRES6-, 4C12-, or palivizumab-treated (control) monkeys. (A) Numbers in the figure inserts are monkey designations. The dotted line indicates the lower limit of detection. The column “group comparison” gives the mean of the log-transformed RNA copies for each group. Numbers in the colors of the different groups indicate fold reduction of the geometric mean of the area under the viral load curve compared to the control group. Kruskal-Wallis tests followed by Dunn’s multiple comparison tests were used to analyze the significance of differences in the area under the viral load curve between the control group and the TRES6 and 4C12 groups, respectively. * indicates a significant (*P* < 0.05) difference in the area under the curve to the control group. (B) Viral RNA load in the indicated lung lobes of the TRES6-, 4C12-, and palivizumab-treated (control) animals.

Two groups of four monkeys each were then injected intravenously with 25 mg/kg body weight of the antibodies TRES6 and 4C12. As a control group, four additional animals received the same dose of palivizumab, a monoclonal antibody targeting the fusion protein of the respiratory syncytial virus (RSV). Palivizumab is approved as a prophylactic medication for high-risk patients, primarily infants, before the winter season (10). Four days later, all monkeys were challenged with 10^5^ TCID50 of the Alpha variant by naso-oropharyngeal spray delivery as described above. In the control group, the viral RNA load in nasal and throat swabs peaked at day 2 after challenge with geometric means of 10^8.4^ and 10^8.0^ copies/mL, respectively (Fig. 1A). TRES6- and 4C12-treated animals’ geometric mean viral RNA levels at day 2 were approximately 100- to 1,000-fold lower than in the control group. Over the next 5 to 6 days, viral loads declined in the upper respiratory tract samples in all groups. On the day of necropsy, geometric means were approximately 100-fold lower in the antibody-treated groups than in the control group. Area under the curve analyses revealed 58- to 132-fold lower viral load levels in the upper respiratory tract samples of the TRES6-treated group than in the control group during the first 6 days after the challenge (Fig. 1). Viral load in the throat and nasal swabs of the 4C12 group was reduced by a factor of 15 and 27, respectively (Fig. 1A).

Different kinetics of the viral load were observed in the lower respiratory tract. In the control group, the geometric mean of the viral loads in the BALF peaked with 10^6.2^ copies/mL at the time of necropsy (Fig. 1A). On day 3, the geometric mean of the viral RNA load was approximately 10,000-fold lower in the antibody-treated groups than in the control group, indicating efficient control of virus spread into the lower respiratory tract. However, in the TRES6-treated animals, an increase of viral load levels in the BALF was observed peaking at the time of necropsy. Looking at the viral loads of individual monkeys revealed viral load levels similar to the control animals in two of the four TRES6-treated monkeys. In contrast, all 4C12-treated animals maintained low viral load levels in the BALF until the time of necropsy, and the viral load, as assessed by area under the curve analysis, was reduced more than 10,000-fold compared to the control group. Consistently, viral RNA loads were generally high in the different lobes of the lungs of two TRES6-treated animals and of all animals of the control group (Fig. 1B). Viral RNA could only be detected in one lobe of the lung of two of the 4C12-treated animals.

**Fig 2:**
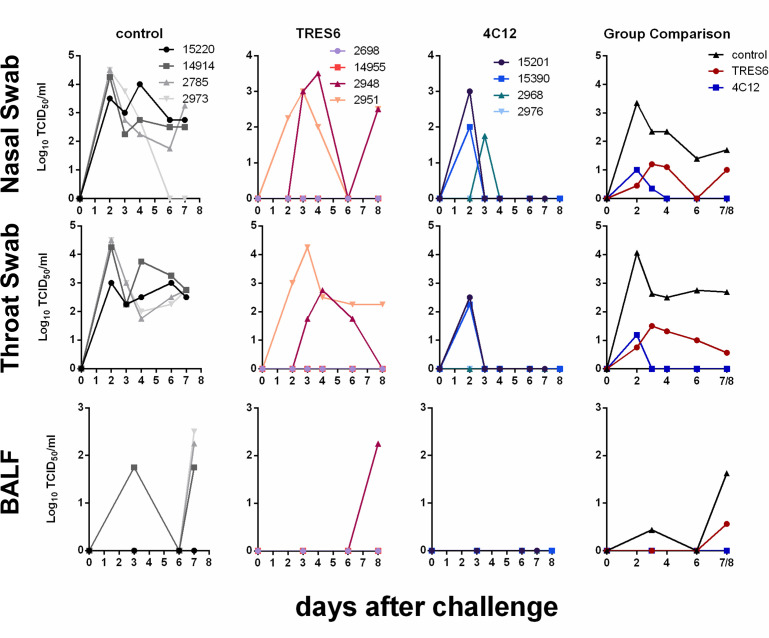
Infectious titers in respiratory secretions of TRES6-, 4C12-, or palivizumab-treated (control) monkeys. Numbers in the figure inserts are monkey designations. Numbers in the colors of the different groups in the group comparisons indicate fold reduction of the geometric mean of the area under the viral load curve compared to the control group.

To assess the effect of the antibodies on infectivity, the infectious titers in respiratory samples were determined at different time points after challenge. Virus could be recovered readily from throat swabs from all control animals at all time points analyzed (Fig. 2). Virus titers were similarly high in nasal swab samples. Pre-exposure prophylaxis with either antibody reduced viral load levels in the upper respiratory tract 51- to 170-fold as assessed by area under the curve analyses on day 6 (Fig. 2). In the BALF samples, virus could only be isolated from one control animal prior to necropsy and from three of the four control animals at necropsy (Fig. 2). With the exception of one animal from the TRES6 group, no virus could be isolated from BALF samples of animals treated with TRES6 or 4C12 (Fig. 2).

**Fig 3:**
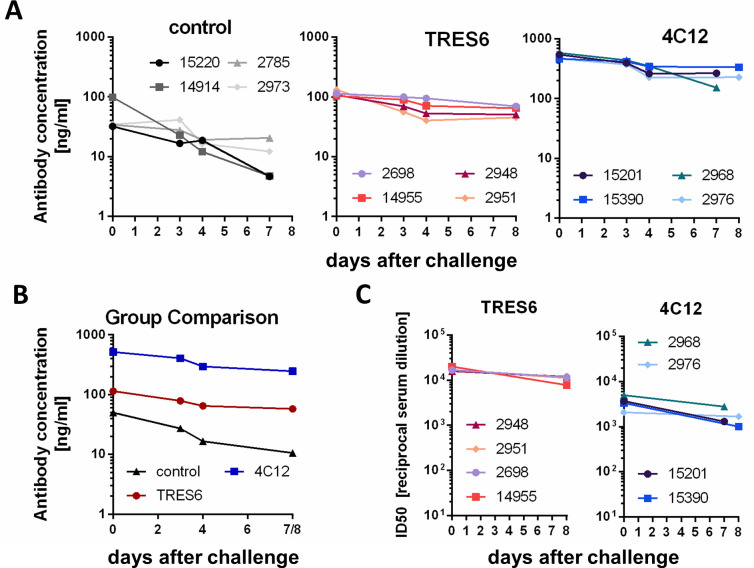
Serum antibody concentrations and neutralization titers. (**A**) Concentrations of administered antibodies in individual monkeys at the indicated days after challenge. Sera from palivizumab-treated (control) animals were assessed for binding to the respiratory syncytial virus (RSV). All other sera were assessed for RBD binding. (**B**) Mean serum concentrations of administered antibodies directed against RBD (4C12, TRES6) or RSV (palivizumab) of the groups indicated. (**C**) Highest reciprocal dilution of sera inhibiting reporter gene activity of lentiviral vectors pseudotyped with the Spike protein of the Alpha variant by >50%. The mean of three independent experiments is shown

The lower viral RNA levels in the BALF samples of the 4C12-treated animals in comparison to those animals treated with TRES6 (Fig. 1) were unexpected since the IC50 of 4C12 against the challenge virus was almost 10-fold higher than for TRES6. On the other hand, 4C12 contains the YTE mutation in the Fc domain conferring increased serum half-life which could lead to potentially higher serum antibody concentrations. Indeed, at the time point of challenge, the 4C12 concentrations in the sera were approximately 400 µg/mL and thus fourfold higher than those of TRES6 (Fig. 3A and B). However, consistent with the higher IC50 of 4C12, the neutralization titer of sera from TRES6-treated animals was higher than those of 4C12-treated animals (Fig. 3C). This argues against the hypothesis that the higher 4C12 antibody concentrations in the sera are responsible for better control of the challenge virus in the lower respiratory tract.

**Fig 4:**
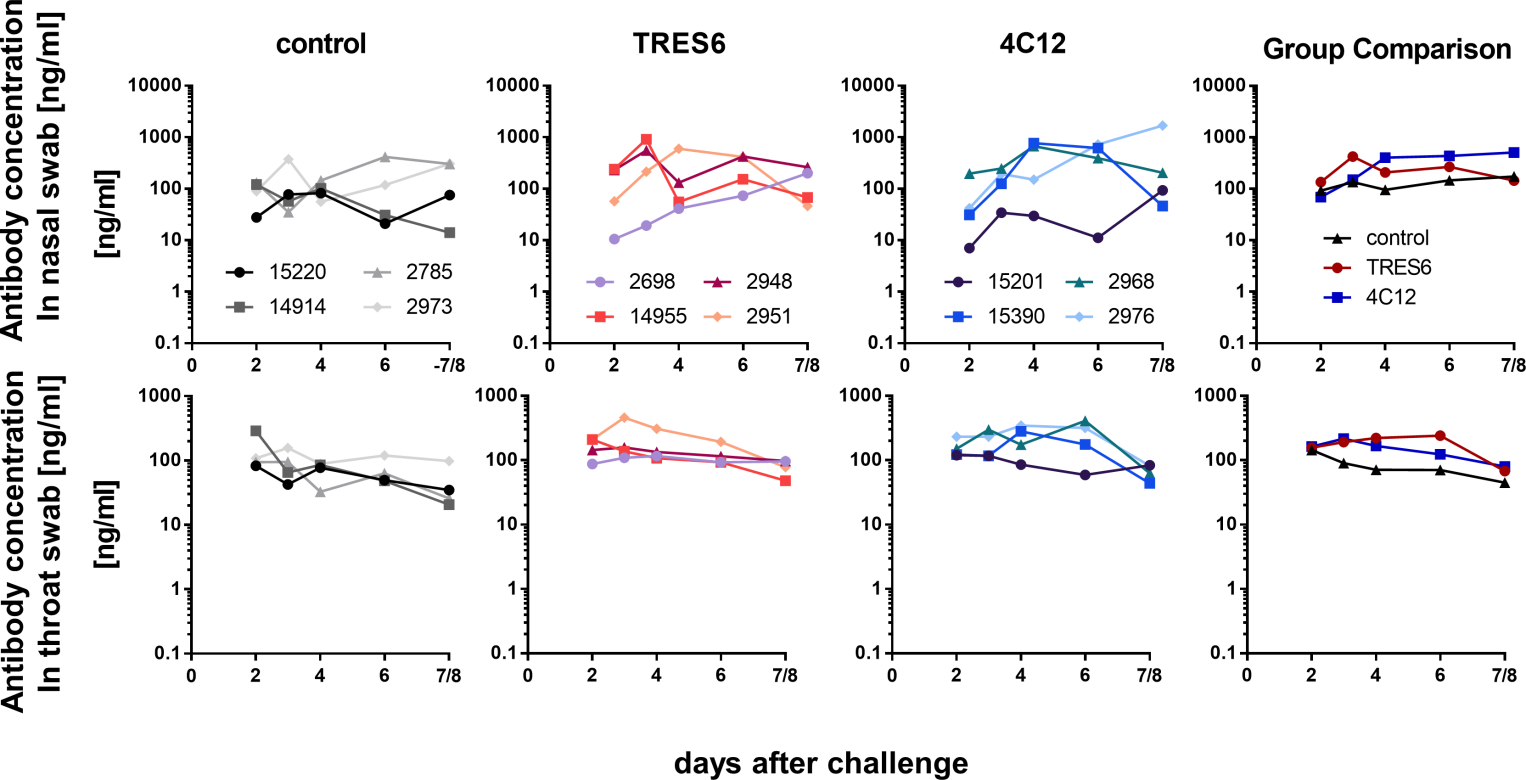
Antibody concentrations in the upper respiratory tract. Concentrations of administered antibodies in eluates of nasal (upper panel) and throat (lower panel) swabs at the indicated days after challenge are shown for each animal and as mean of the groups. The lower limit of detection of the antibodies is 0.12 ng/mL. Sera from palivizumab-treated (control) animals were assessed for binding to respiratory syncytial virus (RSV). All other sera were assessed for RBD binding.

To explore potential differences in the secretion of the antibodies into the respiratory tract, their concentration was determined in nasal and throat swabs and in BALF and lung tissue samples. TRES6 and 4C12 antibodies were readily detectable in the swabs of the upper respiratory tract. Although there was some variation in the antibody levels observed in nasal swabs, 4C12 and TRES6 antibody concentrations in the throat swab eluates were similar and around 100 ng/mL (Fig. 4). Since serum levels of 4C12 were fourfold higher, a smaller proportion of the 4C12 antibodies seemed to reach the mucosal surfaces. While a clear increase in antibody levels in nasal swabs could be observed from day 2 after challenge until the day of necropsy in some animals, 4C12 and TRES6 antibody concentrations in the throat swab samples remained rather constant throughout the observation period.

**Fig 5:**
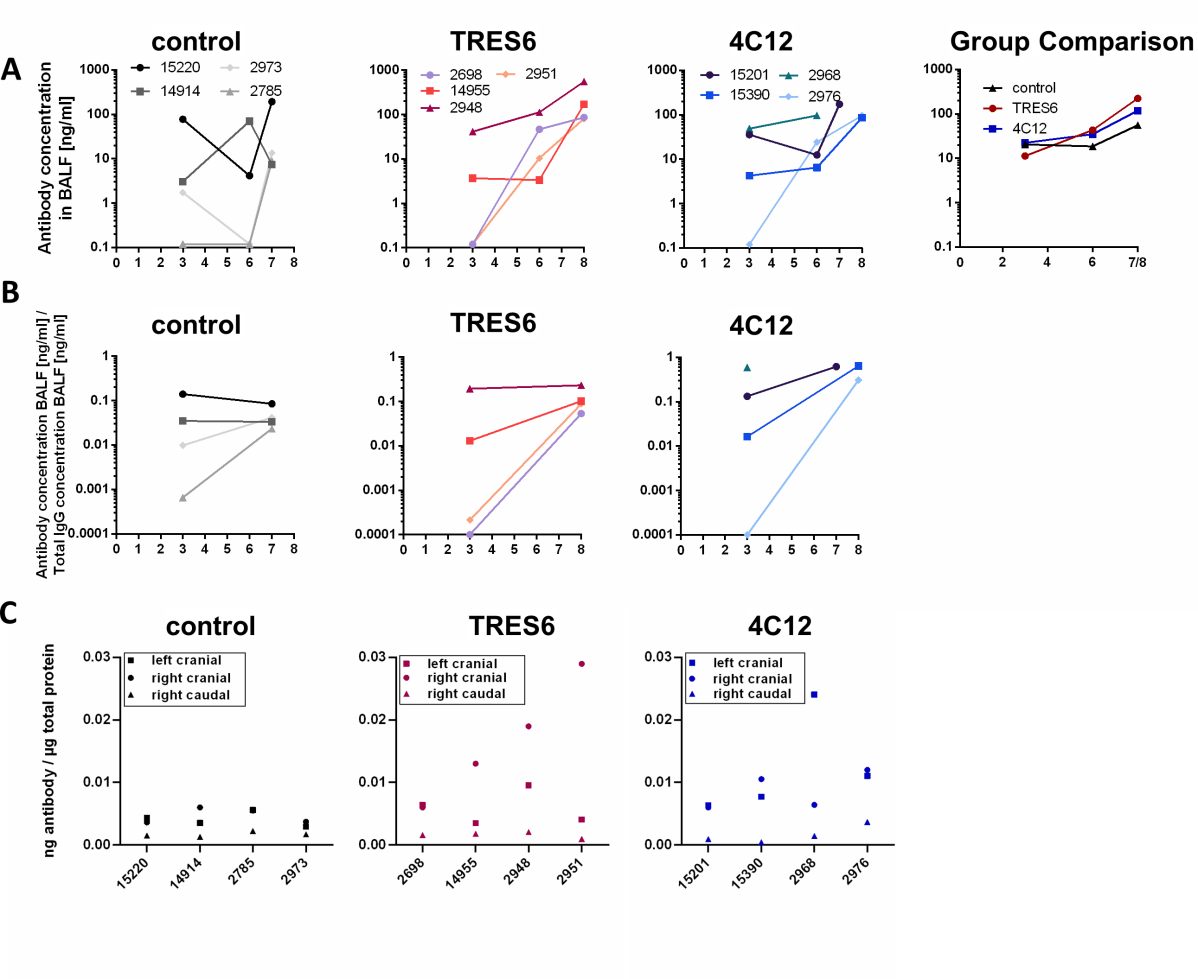
Antibody concentrations in the lower respiratory tract. (**A**) Concentration of administered antibodies in BALF samples of individual monkeys. The panel “group comparison” gives the mean antibody concentration of the groups indicated. (**B**) Ratio of antibody concentrations in BALF samples to the antibody concentrations in sera of individual monkeys at the indicated days after challenge. (**C**) Amount of administered antibodies per microgram total protein of lysates of tissue samples obtained from the indicated pulmonary lobes for individual monkeys. Numbers in the figure inserts (in A) are monkey designations.

Different kinetics of the administered antibodies were observed in the BALF samples (Fig. 5A). In 4 of the 12 study animals, the administered antibodies could not be detected at day 3 after challenge (representing day 7 after antibody administration). With the exception of a single animal (15201), antibody concentrations increased from day 3 to 6 after challenge, a time point when antibody concentrations were detected in all animals. The ratio of antibody concentrations and total IgG concentrations in BALF also increased over time (Fig. 5B) in 9 of 11 animals, indicating that the increase in the concentration of the administered antibodies in the BALF is not primarily due to an enhanced vascular permeability triggered by the ongoing virus replication. Antibody concentrations in BALF samples were similar in the TRES6 and 4C12 group, despite their fourfold difference in their serum concentrations. At the time of necropsy, the amount of antibody per amount of total protein in the sample was also determined (Fig. 5C). Although there was some variation from tissue to tissue sample within each animal, no major difference was observed between groups. Thus, differences in systemic and mucosal antibody concentrations did not seem to be the underlying reason for the inefficient control of virus spread in two of the four TRES6-treated animals.

**Fig 6:**
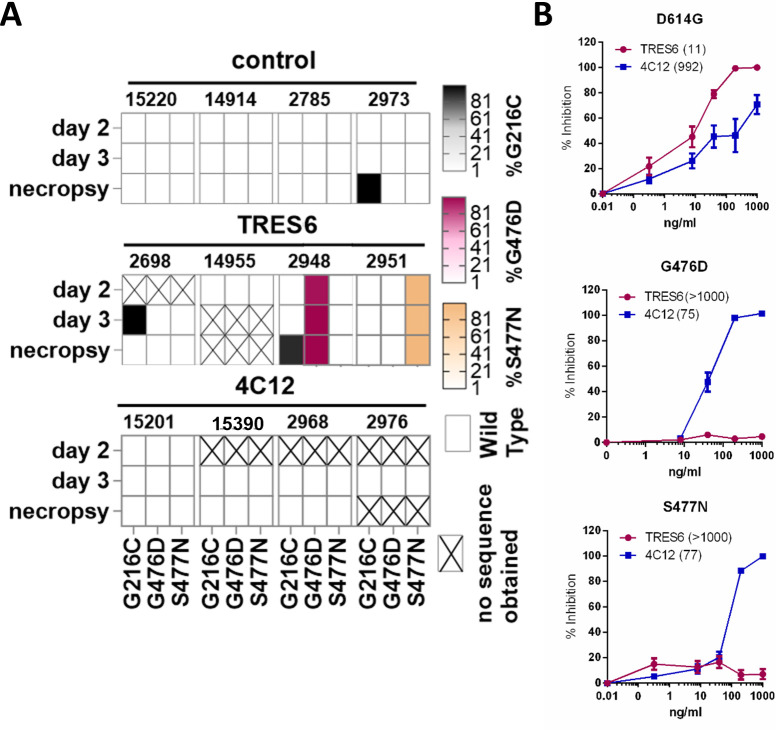
Mutations of the challenge virus and resistance to TRES6 and 4C12. (**A**) The frequency of amino acid mutations in viral amplicons from nasal swab samples was determined by next-generation sequencing. The presence of point mutations that occur at a frequency of >90% of reads is indicated for the different animals and time points after challenge. The sequences were compared to the Alpha (wild type) challenge virus. (**B**) Neutralization of lentiviral particles pseudotyped with the S protein carrying the indicated mutations. Mean and SEM of three experiments performed in triplicate are shown. Numbers in brackets indicate the IC50 in ng/mL.

To explore potential emergence of antibody escape mutants, amplicons from swab samples covering the Spike open reading frame were analyzed by next-generation sequencing, and non-synonymous mutations present in at least one sample in more than 20% of the reads were identified. The point mutations G476D and S477N were consistently detected in the Spike protein of the challenge viruses from the TRES6 animals 2948 and 2951, which displayed high viral load levels in all respiratory fluids. These mutations were already dominant 2 days after challenge at a frequency of 96% and 98% and further increased to nearly 100% on day 3 and the day of autopsy (Fig. 6A). In one animal of the control group, a dominant non-synonymous mutation (G261C) in the Spike protein emerged at the time of autopsy. This mutation was also detected at a frequency of 100% at day 3 of the TRES6 animal 2698 and at necropsy in TRES6 animal 2948, but not at any other samples of these animals or in any other animal with a detection cut-off of 1%. (Fig. 6A). None of the other reading frames of these two animals had dominating non-synonymous mutations. The consistent detection of the G476D and S477N mutation in all samples of one individual TRES6 animal and the absence of the G476D and S477N mutations in the control and 4C12 groups suggests that these mutations emerged within 2 days after infection due to the selective pressure exerted by TRES6. These two mutations could not be detected by next-generation sequencing of the entire genome of the challenge virus stock, although 648 and 658 reads were obtained for the wild-type codons at positions 476 and 477, respectively.

To allow a greater sequencing depth at these positions, amplicons spanning amino acids 424 to 714 of the Spike encoding sequences were generated from the virus stock and a molecular clone of SARS-CoV-2 (11). At a depth of 3,892 reads for the G476 codon of the virus stock, the G476D mutation could not be detected. At a reading depth of 3,898 reads for the S477 codon, the S477N mutation was detectable in four reads. However, in parallel sequencing of a molecular clone of a bacmid containing the SARS-CoV-2 genome encoding G476 of the Spike protein at a comparable reading depth also resulted in two reads encoding G476D and two reads encoding G476V. Similarly, sequencing of position S477 of the bacmid resulted in eight reads deviating from the sequence of the molecular clone, indicating a frequency of PCR or sequencing errors at these two codons in the range of 0.05% to 0.2%. Thus, the G476D and the S477N mutations are either absent in the challenge virus stock or their frequency is below approximately 0.2%, indicating that these mutations either occurred during the first replication cycles in the challenged monkeys or were rapidly selected from a very minor variant of the challenge virus stock.

To determine the effect of the two point mutations on antibody sensitivity, they were introduced into expression plasmids of the Spike protein of the D614G variant. Lentiviral pseudotype assays revealed a strong increase in IC50 values of TRES6 for both mutants to an IC50 higher than 1 µg/mL for S477N and G476D (Fig. 6B). In the 4C12-treated animals, emergence of resistance mutations could not be detected. Therefore, rapid emergence of TRES6 escape mutants of the challenge virus appears to be responsible for the high viral loads observed in two of the four monkeys during the later time points of the observation period (Fig. 1 and 2). Interestingly, virus escape variants against both antibodies could be generated *in vitro* by incubating the virus in the presence of TRES6 or 4C12 for five passages on Vero-E6 cells (Fig. S2). The TRES6 viral escape variant showed a mutation at position P479S. The mutation of this site leads like the previously detected T478 und S477 mutations in TRES6 escape variants, to alterations in the Y473–Y489 loop. This leads to an impaired antibody-RBD interaction (Fig. S3A) (4, 12). Surprisingly, the 4C12 resistant virus variant displayed a mutation at position P621 instead of the expected mutation at the Spike-4C12 interface located in the proximity of residue I468 (Fig. S3B). The mutation at position P621, however, could lead to a stabilization of the closed Spike conformation. In this case, the RBD is presented in a down conformation, thereby interacting in *trans* with the N-terminal domain (NTD) of the neighboring protomer leading to a concealed 4C12 binding site (Fig. S3C). Since this Spike conformation also conceals the RBD, making it less accessible for ACE-2 binding, virus variants carrying this mutation might have a fitness disadvantage at low ACE-2 expression levels. Consistently, variants expressing mutations at position P621 are rarely detected *in vivo* (13). Interestingly, this fitness disadvantage does not seem to affect the virus growth *in vitro*, as shown in (Fig. S2F and G). Here Vero-E6 cells were infected with the same multiplicity of infection (MOI) of either the TRES6 escape variant, the 4C12 escape variant, or a control. Surprisingly, the 4C12 escape variant displayed a similar viral growth kinetic as the TRES6 escape variant or control virus. This indicates that SARS-CoV-2 infection *in vitro* and *in vivo* may differ, mainly due to the high complexity of organisms and the lack of immune pressure in cell culture models (14).

## DISCUSSION

The study clearly demonstrates reduction of viral load and infectivity of the SARS-CoV-2 Alpha variant by TRES6 and 4C12 in a pre-exposure prophylactic nonhuman primate model and thus broadens the spectrum of monoclonal antibodies with confirmed efficacy in nonhuman primates (15–22). Given the differences in the experimental set-up between the different studies, ranking of the different antibodies with regard to *in vivo* potency seems only be possible in side-by-side experiments. However, our study provides three conclusions that are important for pre-exposure prophylaxis of SARS-CoV-2 and other respiratory viral infections by monoclonal antibodies in general.

First, antibody-resistant variants may emerge within 2 days after exposure. This implies that the genetic barrier to resistance should be an important parameter for selection of mAb for prophylaxis and treatment of SARS-CoV-2 and other respiratory viral infections. Evidence for escape of SARS-CoV-2 from neutralization by mAbs has been obtained in COVID-19 patients treated with anti-SARS-CoV-2 antibodies. This was primarily seen when a single therapeutic antibody, like sotrovimab, was administered. Viral escape mutations against this mAb were detectable for the SARS-CoV-2 Omicron BA.1 or Delta Variant, as early on as 3 or 6 days after start of the treatment, respectively (23, 24). Mutations against administered mAbs have also been described to arise during the treatment of infections with the Alpha VOC (25). In addition, given the correlation of neutralizing antibody titers with vaccine efficacy (26), it seems obvious that pre-exposure prophylaxis with mAbs does not protect from infection with variants that are not neutralized by these mAbs. However, whether antibody escape mutants emerge *de novo* after exposure to a sensitive virus when neutralizing antibodies are administered as pre-exposure prophylaxis is more difficult to assess in humans, since the amino acid sequence of the virus individuals under pre-exposure prophylaxis are exposed to is rarely known. While this question can be addressed in animal models, we are aware of only one NHP study analyzing viruses from the challenged monkeys by next-generation sequencing. Similar to our results with 4C12, this study did not find evidence for emergence of viral escape mutants (15). Although we could not detect the TRES6 escape mutations in our challenge virus stock, we cannot exclude that the TRES6 escape mutants are already present in our challenge stock at a frequency below 0.2% and are enriched after infection by the selective pressure exerted by the TRES6 antibody. However, the emergence of two different escape mutants in the two monkeys of the TRES6 group with high viral loads and the absence of these escape mutants in the other two animals indicate that if the two mutants would have been already present in our challenge stock, their frequency must have been below one monkey infectious dose per 10^5^ TCID50. Why we did not see emergence of escape mutants to 4C12 in the challenged monkeys is unclear, since we were able to generate a viral escape variant to 4C12 *in vitro* to the challenge Alpha virus within the same timeframe as viral escape variants against TRES6 arose [for mutations and neutralization assays, see Fig. S2 and (12)]. This could indicate that viral mutations that are tolerable for SARS-CoV-2 *in vitro* differ from those tolerable *in vivo*, underlining the need and usage of animal models for the infection with SARS-CoV-2. Alternatively, a reduced selective pressure of 4C12 compared to TRES6 treatment could delay the emergence of 4C12 escape mutants. However, strongly reduced viral load levels compared to the control group and the absence of drug resistance mutations in monkeys from the 4C12 group argue against this hypothesis.

Second, monoclonal antibodies harboring mutations in the Fc fragment conferring an extended serum half-life (9) may not necessarily increase antibody concentrations in respiratory secretions. Although serum concentrations of 4C12 containing the half-life extending YTE mutations were approximately fourfold higher than for TRES6 (Fig. 3), levels of both antibodies were similar in respiratory tract secretions (Fig. 4). This may be a direct consequence of the enhanced affinity of half-life extended antibody mutants to the FcRn receptor. Thus, higher serum concentrations of half-life extended antibodies may not necessarily improve sterilizing immunity at mucosal sites of viral entry. The YTE mutation has also been shown to enhance the risk of emergence of anti-drug antibodies (27). Better control of pulmonary viral load by the YTE-containing 4C12 antibody, high 4C12 serum concentrations, and a short observation period of 11 to 12 days after antibody administration argue against an effect of YTE-triggered anti-drug antibodies on the efficacy end points of our study.

Third, the biodistribution of intravenously administered mAbs seems to follow different kinetics in the upper and lower respiratory tract. While the antibody concentrations in oropharyngeal secretions have already reached their peak 6 days after intravenous injection, antibody concentrations in BALF samples increase at least until 10 to 11 days after injection. In a subset of animals, the antibodies are not even detectable in the BALF 7 days after injection. Similar kinetics of the control antibody palivizumab in BALF and the absence of detectable Spike-specific IgG levels in BALF of the control group indicate that the observed increase in the concentrations of TRES6 and 4C12 in BALF is not due to autochthonous antibody production during the first week of infection with the challenge virus. To control for changes in vascular permeability by the SARS-CoV-2 infection and variation in sampling of BALF, we also normalized the concentrations of the administered antibodies in BALF for total BALF IgG levels, essentially confirming the initial observation (Fig. 5). However, the kinetics we observed differ from the study by Jones et al., in which the concentrations of the administered antibody in BALF were in a similar range 1 to 6 days after administration (16). Currently, we cannot explain these differences, but delayed distribution of intravenously injected antibodies to vaginal surfaces (28) and rectal fluid (15) constitute interesting precedent cases. Whether a delay of antibody penetration into lower respiratory tract secretions impairs the level of protection from lower respiratory tract infections remains to be determined and could potentially support the exploration of alternative administration routes of the antibodies. The prophylactic intranasal applications of anti-SARS-CoV-2 mAbs has, for example, reduced lung pathology in a SARS-CoV-2 mouse model (29). Administration of mAbs or vectors encoding them via the respiratory tract could therefore potentially also improve mAb levels in human lungs.

## MATERIALS AND METHODS

### Virus propagation and neutralization assay

The SARS-CoV-2 Alpha Virus variant (B.1.1.7) was propagated as previously described (4). Virus neutralization assays were conducted as described earlier. To this end, serial dilutions of the antibodies were incubated with 1.88 × 10^4^ infectious units of the SARS-CoV-2 Alpha VOC for 1 h. The mix was then added onto 2 × 10^4^ Vero-E6 cells seeded per well of a 96-well plate (Greiner, Kremsmünster, Austria) on the day prior to the infection. After a change to fresh OptiPRO (Thermo Fisher, Waltham, USA) culture medium, the cells were incubated for 24 h. After removal of the supernatant, cells were washed and fixed and permeabilized for 20 min with 4% paraformaldehyde (PFA). After blocking with 5% skimmed milk for 1 h, the cells were stained with an antibody mix of murine origin that recognizes SARS-CoV-2 and a secondary fluorescein isothiocyanate (FITC) -conjugated goat anti-mouse IgG serum (Jackson ImmunoResearch #115-095-003) (4). The plates were read out with a CTL-ELISPOT reader (Immunospot; CTL Europe GmbH, Bonn, Germany). Signal analysis was performed with the ImmunoSpot fluoro-X suite (Cellular Technology Limited, Cleveland, USA). By plotting the percent virus activity against the antibody concentration and usage of the normalized response vs inhibitor formula (variable slope) of GraphPad Prism (San Diego, USA), the IC50s were calculated.

### Preparation and characterization of the challenge virus

In addition, 5.5 × 10^4^ Calu-3 cells per cm^2^ were seeded 48 h prior to the infection in Dulbecco's Modified Eagle Medium (DMEM) supplemented with 4.5 g/L glucose, 10% fetal calf serum (FCS), and 1% Pen/Strep (all PAN-Biotech, Aidenbach, Germany) into 75 cm^2^ flasks. On the day of infection, the medium was discarded, and after a washing step with 2 mL DMEM supplemented with 4.5 g/L Glucose, 2% FCS and 1% penicillin/streptomycin 270 µL SARS-CoV-2 strain MUC-IMB-CB B1.1.7 (TCID50 1.08 × 10^6^, GISAID EPI_ISL_755639) were added onto the cells diluted in 3 mL 2% FCS medium. The virus was left to adsorb to the cells for 90 min before 10 mL 2% FCS medium were added. Following an incubation period of 3 days or after cytopathic effects were visible, 10 mL of the supernatant was centrifuged at 1,200 rpm for 10 min. In parallel, the flasks containing the adherent cells and the remaining medium were transferred to −80°C until the liquid was frozen. Cells and supernatant were subsequently thawed and transferred into tubes and centrifuged for 15 min at 1,200 rpm. After pooling of the virus containing supernatants, they were sterile filtered through a 0.2 µm filter and stored at −80°C until needed. The TCID50 was determined by application of serially diluted virus onto Calu-3 cells that were seeded into 48-well plates at a density of 120,000 cells per well 48 h prior to the titration. Seventy-two to 96 h after the infection, the cytopathic effect was quantified, and the titer was calculated according to Reed and Muench (30). Variant sequence identity was confirmed by amplicon sequencing as described previously (4, 12).

### Antibody production

TRES6 and 4C12 were produced in CHO cells and purified by affinity chromatography (Biointron, Metuchen, USA). Palivizumab (Synagis, Waltham, USA) was produced by Boehringer Ingelheim (Ingelheim am Rhein, Germany) and purchased from AbbVie (North Chicago, USA).

### Nonhuman primate study

#### Ethical statement for animal experiments

Five- to 12-year-old purpose bread rhesus macaques (*Macaca mulatta*), eight females and six males with a body weight between 4.8 kg and 10.0 kg, were obtained from the DPZ breeding colony. The animals were kept either in groups of two, if socially compatible, or in individual cages, then with constant visual, olfactory, and acoustic contact to their roommates. If kept individually, animals were still able to groom their neighbors through small mash inserts in the side walls of their cages. The cages contained perches and environmental enrichments. Continuous access to water was ensured and feeding took place twice daily with dry monkey biscuits containing adequate carbohydrates, fats, fibers (10%), minerals, proteins, and vitamins. Additional edibles like fresh fruits, vegetables, nuts, cereal pulp, and seeds were also provided. During the study, animals were scored by experienced keepers for any signs of suffering, pain, or illness by checking feed and water intake, stool consistency, and general condition twice a day. In case of any abnormalities, animals were attended by veterinarians of the institute.

#### Experimental animals, specimen collection, antibody treatment, and virus inoculation

Overall, 14 rhesus monkeys (for details, see Ethical statement) were assigned to the study. Two females were used for viral dose finding. The other animals were randomly allocated to three groups with four monkeys each considering equal distribution of the sexes. For all interventions, animals were anesthetized by intramuscular injection of a mixture 5–10 mg ketamine, 1–2 mg xylazine, and 0.01–0.02 mg atropine per kilogram body weight. Bleeding was done twice prior to antibody and/or virus inoculation and repeatedly after viral challenge exposure until necropsy by puncture of the femoral vein employing the Vacutainer system (BD). In addition, nasal and throat swabs were collected in 1 mL virus transport medium (VTM, Mast Diagnostica, Germany). For the nasal sample, an individual swab (FLOQSwab Minitip, COPAN, Italy) was inserted into each nostril, 10 times carefully rotated, both swabs reconstituted in one tube with 1 mL VTM, swirled, and immediately stored on ice. Throat secretion was obtained by wiping the throat with two separate swabs (FLOQSwab regular, COPAN, Italy) which were then reconstituted each in 1 mL VTM, stored on ice, and the eluate pooled later on. BALF was collected by bronchoscopy once or twice before any viral or antibody intervention and 3, 6, and 7 or 8 days after viral challenge. For that purpose, the bronchoscope was inserted into the trachea and further slid into a bronchus to wedge position. Then, 3 mL of pre-warmed 0.9% NaCl was injected and immediately aspirated. BALF recovery varied between 0.5 mL and 1.5 mL, and the fluid was immediately stored on ice.

Administration of the antibodies was performed intravenously by bolus injection of 10–15 mL into the right or left saphenous vein at a dose of 25 mg/kg body weight. Four days after antibody treatment, animals were challenged with a 10^5^ TCID50 of SARS-CoV-2 Alpha through intranasal and oropharyngeal spray application with CE-marked, small, disposable, mechanic aerosolization devices attached to a 1 mL syringe (MAD nasal device and MADgic drug atomization device for children, Teleflex). First, 750 µL of virus suspension was nebulized in the throat followed by nebulization of 250 µL virus suspension into each nostril. Before the virus was administered to the throat and nose, the lung was blocked by a cuff of the endotracheal tube that was left inserted in the trachea for 20 min after virus administration to limit the initial infection to the upper respiratory tract. The two dose-finding animals received the virus without any antibody treatment. At necropsy on day 7 or 8 after viral challenge, animals were anesthetized with 10 mg ketamine, 2 mg xylazine, and 0.02 mg atropine per kilogram body weight and euthanized by an overdose of 160–240 mg sodium pentobarbital per kg body weight injected into the circulation. At necropsy, a variety of tissues were collected with particular emphasis on the six different lung lobes. Each tissue piece was stored on ice after removal and transferred to −80°C within 2 h until further use or fixed in formaldehyde (see below).

#### Determination of viral RNA levels

Viral RNA was isolated from 140 µL thawed swab or BALF samples following the QIAamp Viral RNA Mini Kit protocol (Qiagen, Hilden, Germany). For qRT-PCR, 8.5 µL purified SARS-CoV-2 RNA-containing solution was reverse transcribed and amplified using TaKaRa PrimeScript One-Step RT-PCR Kit (TaKaRa Bio Europe, Goteborg, Sweden). Reverse transcription and amplification were performed as described (31), employing the Rotor-Gene Q apparatus and software (Qiagen) as described by the supplier. The primer and probe oligonucleotide sequences were as follows: E Sarbeco forward (5´-ACAGGTACGTTAATAGTTAATAGCGT-3´), E Sarbeco reverse (5´-ATATTGCAGCAGTACGCACACA-3´), and a fluorescent probe (5´-(6FAM) ACACTAGCCATCCTTACTGCGCTTCG (BHQ1)−3´), and were purchased from Sigma Aldrich (32). Standard curves were used to calculate RNA copies per milliliter. *In vitro* transcribed serially diluted E gene RNA was used as standard. E gene RNA was kindly provided by Artur Kaul, Infection Biology Unit, German Primate Center. Absolute copy numbers of the standard were determined using Qiacuity Digital PCR System, Qiagen, Hilden, Germany. Quantification was performed by two independent PCR reactions. The detection limit for viral RNA was 42 viral copies per milliliter.

Additionally, viral RNA was analyzed in the six different lung lobes of each animal. A small tissue piece of each lung location of 8.7–68.8 mg was cut-off on dry ice from the frozen tissue block conserved in RNAprotect (Qiagen, Hilden, Germany), weighed, and transferred to a 2 mL tube of Precellys ceramic kit prefilled with 2.8 mm steel beads (VWR, Radnor, USA). Next, 600 µL of 1× DNA/RNA Protection Reagent from the Monarch Total RNA Miniprep Kit (NEB, Ipswich, USA) was added, and the mixture was homogenized in a Precellys 24 tissue homogenizer (Bertin Technologies, Paris, France) for 3 × 20 s with a break of 10 s between each cycle. Because of foam formation, the homogenate was briefly centrifuged by pressing the preinstalled short key for 2 s. Thereafter, purification steps were performed as described in the kit manual. RNA was eluted with 50 µL of RNase-free water and stored at −80°C until further use. The RT-digital (d)PCR procedure was carried out following the manufacturer´s instructions using the QIAcuity One, 2plex device (Qiagen, Hilden, Germany), the QIAcuity One-Step Viral RT-PCR kit (Qiagen, Hilden, Germany), and the 24-well 26 Nanoplates (Qiagen, Germany). In brief, the RT-dPCR reaction mixture consisted of 10 µL 4× One-Step Viral RT-PCR Master Mix, 0.4 µL 100× Multiplex Reverse Transcription Mix, 0.5 µL of each primer (see above, 10 pmol) and 0.3 µL probe (see above, 10 pmol), 8.5 µL of extracted RNA, and 19.8 µL RNase-free water in a final volume of 40 µL. The amplification protocol included 50°C for 30 min for reverse transcription, 58°C for 10 min for RT enzyme inactivation, 95°C for 2 min for enzyme activation, and 40 two-step cycles with 5 s at 95°C and 30 s at 60°C. All reactions included a positive control (diluted standard) and a negative control (nuclease-free water). Quantification was performed by two independent PCR reactions and the data were analyzed using the QIAcuity Software Suite 2.0.20 (Qiagen, Hilden, Germany). Copy numbers were normalized to 1 g of tissue. Depending on the amount of tissue used for RNA extraction, the limit of detection varied from 87 to 678 copies per gram tissue.

#### Determination of infectious titers

In addition, the viral titers in the eluates and the BALF were measured. Here, 120,000 Calu-3 cells were seeded in DMEM supplemented with 4.5 g/L glucose, 10% FCS, and 1% Pen/Strep (all PAN-Biotech, Aidenbach, Germany) per well into a 48-well plate 48 h before the infection. On the day when the respiratory fluids were added, the medium was changed to 400 µL DMEM supplemented with 5 g/L glucose, 2% FCS, and 1% Pen/Strep. Thereafter, serial dilutions, ranging from 10^−1^- 10^−4^, of nasal or throat swab eluate or BALF were added in quadruplicates. Seventy-two to 96 h after the infection, the cytopathic effect was quantified, and the titer was calculated according to Read and Muench.

#### Determination of antibody concentrations in NHP samples

To determine the concentrations of the administered antibodies in swabs, sera, BALF, or organ samples, microtiter plates (Greiner Bio-One, Kremsmünster, Austria) were coated overnight as described previously with 100 ng/well of RBD (Diarect, Freiburg im Breisgau, Germany, #46100) or 5 × 10^5^ inactivated plaque-forming units (PFU) of RSV (33, 34). After blocking eluted swab, sera, BALF, and lysed organ samples were incubated for 1 h. Additionally, a serial dilution of either TRES6, 4C12, or palivizumab was applied as a standard. Following a washing step, a goat anti-human horse radish peroxidase (HRP) -coupled (Dianova, Hamburg, Germany) antibody was applied and incubated for 1 h. Subsequently, the plates were washed and the relative light units were detected with a multilabel plate reader Victor X4 (Perkin Elmer, Hamburg, Germany). Afterward, the absolute antibody content was calculated by interpolation against the standard curve with GraphPad PRISM 6 (San Diego, USA). The total IgG content of samples was analyzed with a Human IgG ELISA BASIC kit (Mabtech, Nacka Strand, Sweden). The assay was performed according to the manufacturer’s recommendations.

#### Analysis of antibody neutralization using a lentiviral pseudotype assay

Antibody neutralization was analyzed as described previously using a lentiviral pseudotype neutralization assay (12, 35–38). Briefly, ACE-2 overexpressing HEK293T cells was infected in the presence or absence of serially diluted sera with lentiviral vector particles produced in HEK293T cells (ECACC 12022001), pseudotyped with the wild-type D614G Spike protein, its G476D or S477N mutants, or the Spike of the Alpha VOC, and transferring a luciferase reporter gene (11, 39). Forty-eight hours after infection, the cells were washed and lysed with ONE-Glo (Promega, Madison, USA), and the bioluminescence signal was assessed on a multilabel plate reader Victor X4. The 50% inhibitory dilution of monkey sera and the IC50 of monoclonal antibodies were calculated with the Sigmoidal 4PL function of GraphPad prism 9.

#### Preparation of lung extracts

Lung samples were obtained at necropsy and stored in cryotubes at −80°C. Frozen samples were lysed in 1 mL of cell extraction buffer, containing 100 nM Tris, pH7.4, 150 mM NaCl, 1 mM EGTA, 1 mM EDTA, 1% Triton X-100, 0.5% sodium deoxycholate (all Carl Roth, Karlsruhe, Germany), and the complete protease inhibitor cocktail (Roche, Basel, Switzerland) in a gentleMACS Dissociator (Miltenyi, Bergisch Gladbach, Germany). The homogenate was kept under agitation for 2 h on an orbital shaker at 4°C. Following a centrifugation step at 13,000 rpm for 20 min, the supernatant was aliquoted into fresh tubes and stored at −80°C until needed. Additionally, the total amount of protein in the lung lysates was assessed with a Pierce BCA Protein Assay kit (Thermo Fisher, Waltham, USA) according to the manufacturer’s instructions.

#### Viral sequence analysis

Isolated RNA from rhesus macaques, at time points with a high viral load, were sequenced for the detection of escape mutations. To this end, the NEBNext ARTIC SARS-CoV-2 Library Prep Kit (Illumina) (NEB, Ipswich, USA) was employed. The protocol recommended by the manufacturer was followed except that the kit was supplemented with additional primers so that a better coverage could be achieved; the primer sequences are available upon request. The samples were read with a MiSeq Reagent Kit v2 (500 Cycles) on an Illumina MiSeq instrument (San Diego, USA) and analyzed with the CLC Genomic Workbench 21 (Qiagen, Aarhus, Denmark).

## Data Availability

The data generated or analyzed during this study are included in this article or available upon request. The RNAseq data can be accessed under Bioproject PRJNA1115950 and Biosample SAMN41518114.

## SUPPLEMENTAL MATERIAL

Supplemental material jvi.00628-24-s0001.docx Supplemental methods; Figures S1 to S3.

ASM does not own the copyrights to Supplemental Material that may be linked to, or accessed through, an article. The authors have granted ASM a non-exclusive, world-wide license to publish the Supplemental Material files. Please contact the corresponding author directly for reuse.
