# Supplementary material for: Genetic barrier to resistance: a critical parameter for efficacy of neutralizing monoclonal antibodies against SARS-CoV-2 in a nonhuman primate model
Source: J Virol. 2024 Jun 20;98(7):e00628-24. doi: 10.1128/jvi.00628-24 (PMC11265388; doi:10.1128/jvi.00628-24)
Supplement: Supplemental material — Supplemental methods; Figures S1 to S3. [file jvi.00628-24-s0001.docx]

**Supplement**

**Genetic barrier to resistance: a critical parameter for efficacy of neutralizing monoclonal antibodies against SARS-CoV-2 in a non-human primate model**

**Supplemental Material and Methods**

**Selection of escape mutants in cell culture**

Escape mutants against TRES6 and 4C12 were generated as described preciously [1]. Briefly, 2 × 10^6^ Vero-E6 cells were seeded into T75 flasks (Greiner, Kremsmünster, Austria) 16-20 hours prior to the infection. The 4C12 or TRES6 antibodies were incubated for 1 hour at 37°C with MUC-IMB-CB B1.1.7 (GISAID EPI_ISL_755639) at a TCID50 of 1 × 10^6^ in 1 ml OptiPRO^TM^. The respective antibodies IC_50_ were used as starting concentration. The antibody concentration was doubled for every round of infection. During the preincubation step the medium was changed in the cell culture flasks to 14 ml fresh OptiPRO^TM^ and afterwards the antibody-virus mixture was added. One additional flask without antibodies was kept as a control. Subsequently the cells were checked daily for cytopathic effects. When those were visible the supernatants were harvested, centrifuged at 1200 rpm for 5 min and filtered through a 0.45 μm filter. Following 100 µl of the supernatant were used for the next round of infection. Five rounds of infection were performed per antibody. Thereafter, the viral isolates were sequenced and mutations identified as described earlier [1, 2].

**Virus growth kinetic**

The escape mutants generated against TRES6 and 4C12 were assessed regarding their fitness in vitro in a virus growth kinetic. To this end 1 × 10^6^ Vero-E6 cells were seeded per well of a 6-well plate 16-20 hours prior to the infection. Following the growth medium was discarded and 2 ml of OptiPRO^TM^ added as well as the same multiplicity of infection (MOI) of either the TRES6 escape variant, the 4C12 escape variant or the control. Following aliquots of the supernatant were taken at 0, 8, 16, 24, 32, 40 and 48 hours post infection. Finally, the C_T_ values of the samples were determined as described previously by Klessing et al 2023 [3, 4].

**Supplemental Figures**


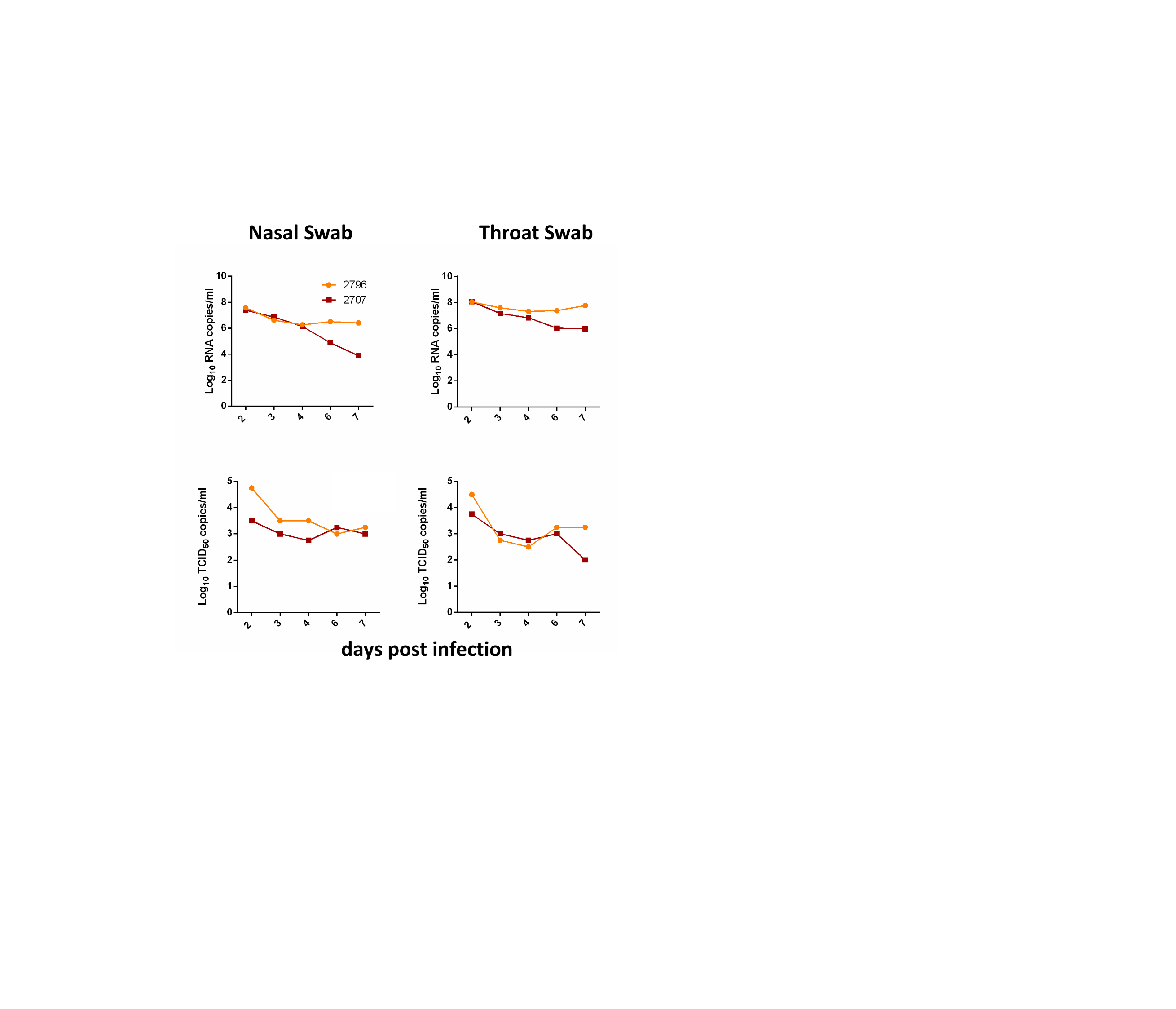


**Supplement Fig 1: Viral RNA load and infectious titers in respiratory secretions of SARS-CoV-2 infected monkeys from the pilot experiment.** Numbers in the figure inserts are monkey designations. Animal 2796 was infected with a 1x10^6^ TCID_50_ and 2707 1x10^5^ TCID_50_ of SARS-CoV-2 Alpha through an intranasal and oropharyngeal spray application. Samples were taken at the indicated days post infection. The animals were sacrificed on day 7.


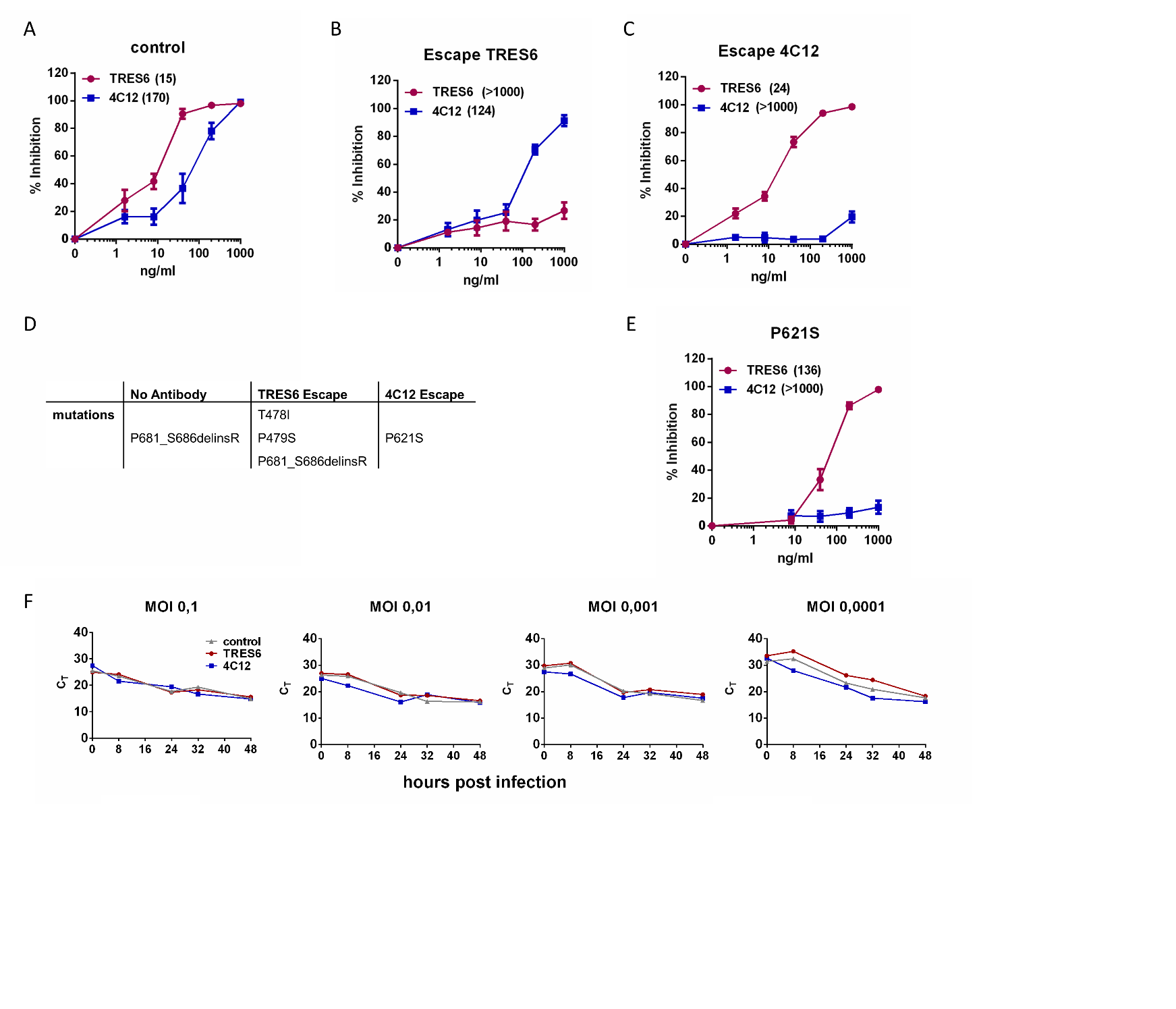


**Supplement Fig 2: Neutralization of escape variants against 4C12 and TRES6, identification of mutations, and in vitro growth kinetics of the escape variants.** A, B, C) Neutralization of a SARS-CoV-2 Alpha variants passaged five times on VeroE6 either without antibody (A), in the presence of TRES6 B) or 4C12 C). The mean of three independent experiments is given. The IC_50_ is indicated in ng/ml in brackets. D) Mutations identified through sequencing in comparison to the Spike protein of Alpha SARS-CoV-2 challenge virus in the viral isolates obtained after 5 passages. E) Percent inhibition mediated by TRES6 or 4C12 of lentiviral particles pesudotyped with P621S. The IC_50_ of the respective antibodies is indicated in brackets. Shown is the mean of three independent experiments. F) C_T_ kinetic of the TRES6 escape variant, 4C12 escape variant or control after the infection of Vero-E6 cells with a MOI of either 0.1, 0.001 or 0.0001. Samples were taken at 0, 8, 16, 24, 32, 40 and 48 hours post infection.


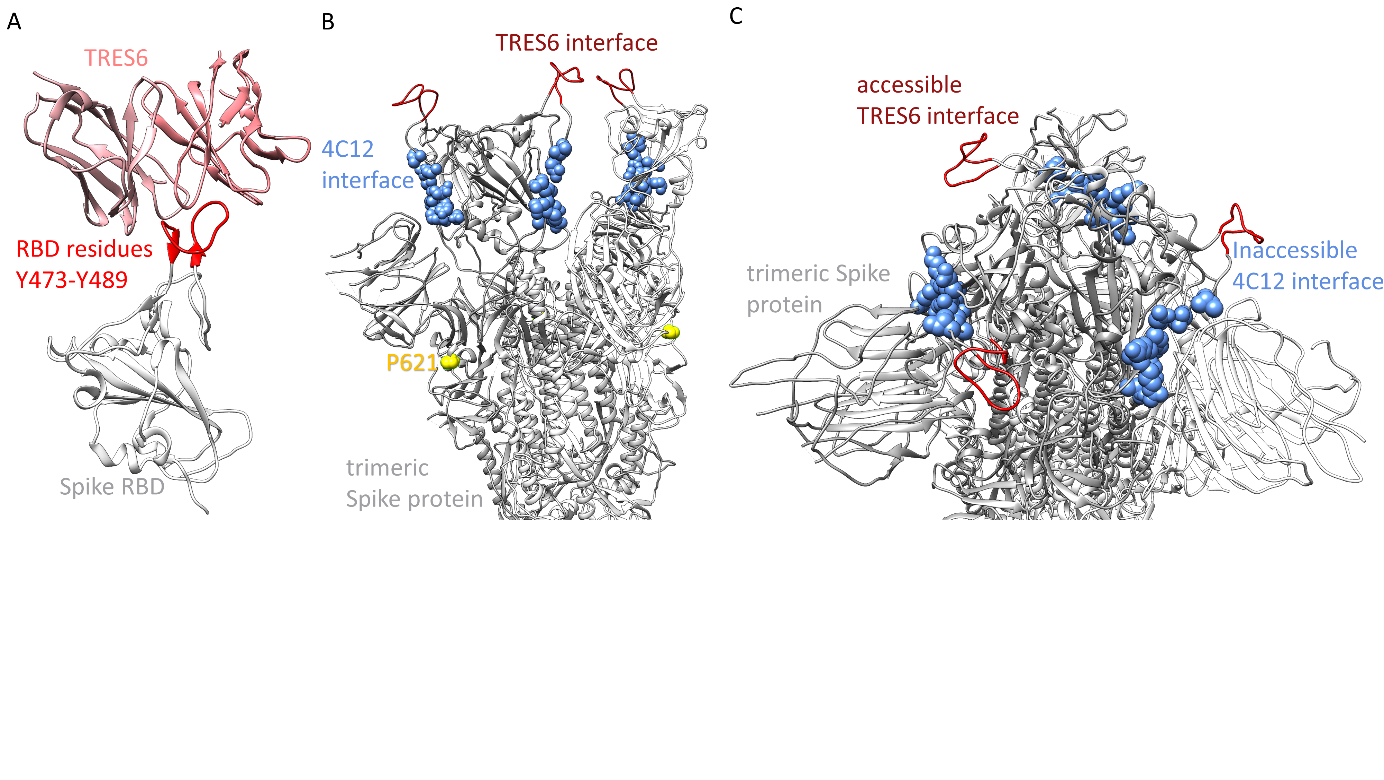


**Supplement Fig 3: Structural modeling of TRES6 bound to the receptor-binding domain (RBD) and comparison of the antibody interfaces for TRES6 and 4C12 on different states of the trimeric SARS-CoV-2 Spike.** A) TRES6 (pink) in complex with Spike RBD (grey). The homology modeling for TRES6 in complex with the RBD based on the structure of the neutralizing antibody Ab4 in complex with the RBD of the SARS-CoV-2 Spike (PDB ID code: 7E39 [5]. B) Position of the TRES6 and 4C12 interfaces on the trimeric Spike protein with all three RBDs in the up conformation (PDB ID code: 7KMS [6]) demonstrating the accessibility of all interfaces. Additionally, the residue P621 is highlighted in yellow. Mutations at this position could lead to a stabilization of the closed Spike conformation thereby concealing the 4C12 interface whilst the TRES6 interface remains accessible, as shown in C) with an trimeric Spike in the closed state (PDB ID code: 7DF3 [7]). The TRES6 interaction interface on the RBD is shown as a red loop whilst the 4C12 interface is shown as blue spheres.

REFERENCES

1. Peter, Antonia S.; Grüner, Eva; Socher, Eileen; Fraedrich, Kirsten; Richel, Elie; Mueller-Schmucker, Sandra; Cordsmeier, Arne; Ensser, Armin; Sticht, Heinrich, and Überla, Klaus, *Characterization of SARS-CoV-2 Escape Mutants to a Pair of Neutralizing Antibodies Targeting the RBD and the NTD.* International Journal of Molecular Sciences, 2022. **23**(15).

2. Peter, Antonia Sophia; Roth, Edith; Schulz, Sebastian R.; Fraedrich, Kirsten; Steinmetz, Tobit; Damm, Dominik; Hauke, Manuela; Richel, Elie; Mueller-Schmucker, Sandra; Habenicht, Katharina; Eberlein, Valentina; Issmail, Leila; Uhlig, Nadja; Dolles, Simon; Grüner, Eva; Peterhoff, David; Ciesek, Sandra; Hoffmann, Markus; Pöhlmann, Stefan; McKay, Paul F.; Shattock, Robin J.; Wölfel, Roman; Socher, Eileen; Wagner, Ralf; Eichler, Jutta; Sticht, Heinrich; Schuh, Wolfgang; Neipel, Frank; Ensser, Armin; Mielenz, Dirk; Tenbusch, Matthias; Winkler, Thomas H.; Grunwald, Thomas; Überla, Klaus, and Jäck, Hans-Martin, *A pair of noncompeting neutralizing human monoclonal antibodies protecting from disease in a SARS-CoV-2 infection model.* European journal of immunology, 2021: p. 10.1002/eji.202149374.

3. Corman, Victor M.; Landt, Olfert; Kaiser, Marco; Molenkamp, Richard; Meijer, Adam; Chu, Daniel Kw; Bleicker, Tobias; Brünink, Sebastian; Schneider, Julia; Schmidt, Marie Luisa; Mulders, Daphne Gjc; Haagmans, Bart L.; van der Veer, Bas; van den Brink, Sharon; Wijsman, Lisa; Goderski, Gabriel; Romette, Jean-Louis; Ellis, Joanna; Zambon, Maria; Peiris, Malik; Goossens, Herman; Reusken, Chantal; Koopmans, Marion Pg, and Drosten, Christian, *Detection of 2019 novel coronavirus (2019-nCoV) by real-time RT-PCR.* Euro surveillance : bulletin Europeen sur les maladies transmissibles = European communicable disease bulletin, 2020. **25**(3): p. 2000045.

4. Klessing, Stephan; Peter, Antonia S.; Fraedrich, Kirsten; Wagner, Jannik T.; Kummer, Mirko; Deutschmann, Janina; Steininger, Philipp; Steibl, Hans-Dieter, and Überla, Klaus *Propagation of SARS-CoV-2 in a Closed Cell Culture Device: Potential GMP Compatible Production Platform for Live-Attenuated Vaccine Candidates under BSL-3 Conditions?* Viruses, 2023. **15**, DOI: 10.3390/v15020397.

5. Nie, Jianhui; Xie, Jingshu; Liu, Shuo; Wu, Jiajing; Liu, Chuan; Li, Jianhui; Liu, Yacui; Wang, Meiyu; Zhao, Huizhen; Zhang, Yabo; Yao, Jiawei; Chen, Lei; Shen, Yuelei; Yang, Yi; Wang, Hong-Wei; Wang, Youchun, and Huang, Weijin, *Three epitope-distinct human antibodies from RenMab mice neutralize SARS-CoV-2 and cooperatively minimize the escape of mutants.* Cell Discovery, 2021. **7**(1): p. 53.

6. Zhou, Tongqing; Tsybovsky, Yaroslav; Gorman, Jason; Rapp, Micah; Cerutti, Gabriele; Chuang, Gwo-Yu; Katsamba, Phinikoula S.; Sampson, Jared M.; Schön, Arne; Bimela, Jude; Boyington, Jeffrey C.; Nazzari, Alexandra; Olia, Adam S.; Shi, Wei; Sastry, Mallika; Stephens, Tyler; Stuckey, Jonathan; Teng, I. Ting; Wang, Pengfei; Wang, Shuishu; Zhang, Baoshan; Friesner, Richard A.; Ho, David D.; Mascola, John R.; Shapiro, Lawrence, and Kwong, Peter D., *Cryo-EM Structures of SARS-CoV-2 Spike without and with ACE2 Reveal a pH-Dependent Switch to Mediate Endosomal Positioning of Receptor-Binding Domains.* Cell Host & Microbe, 2020. **28**(6): p. 867-879.e5.

7. Xu, Cong; Wang, Yanxing; Liu, Caixuan; Zhang, Chao; Han, Wenyu; Hong, Xiaoyu; Wang, Yifan; Hong, Qin; Wang, Shutian; Zhao, Qiaoyu; Wang, Yalei; Yang, Yong; Chen, Kaijian; Zheng, Wei; Kong, Liangliang; Wang, Fangfang; Zuo, Qinyu; Huang, Zhong, and Cong, Yao, *Conformational dynamics of SARS-CoV-2 trimeric spike glycoprotein in complex with receptor ACE2 revealed by cryo-EM.* Science Advances, 2021. **7**(1): p. eabe5575.
